# Thiamethoxam Resistance in the House Fly, *Musca domestica* L.: Current Status, Resistance Selection, Cross-Resistance Potential and Possible Biochemical Mechanisms

**DOI:** 10.1371/journal.pone.0125850

**Published:** 2015-05-04

**Authors:** Hafiz Azhar Ali Khan, Waseem Akram, Javaid Iqbal, Unsar Naeem-Ullah

**Affiliations:** 1 Institute of Agricultural Sciences, University of the Punjab, Lahore, Pakistan; 2 Department of Entomology, University of Agriculture, Faisalabad, Pakistan; 3 University College of Agriculture and Environmental Science, The Islamia University of Bahawalpur, Pakistan; 4 On Farm Water Management Office, Punjab Government, Multan, Pakistan; Fundação Oswaldo Cruz, BRAZIL

## Abstract

The house fly, *Musca domestica* L., is an important ectoparasite with the ability to develop resistance to insecticides used for their control. Thiamethoxam, a neonicotinoid, is a relatively new insecticide and effectively used against house flies with a few reports of resistance around the globe. To understand the status of resistance to thiamethoxam, eight adult house fly strains were evaluated under laboratory conditions. In addition, to assess the risks of resistance development, cross-resistance potential and possible biochemical mechanisms, a field strain of house flies was selected with thiamethoxam in the laboratory. The results revealed that the field strains showed varying level of resistance to thiamethoxam with resistance ratios (RR) at LC_50_ ranged from 7.66-20.13 folds. Continuous selection of the field strain (Thia-SEL) for five generations increased the RR from initial 7.66 fold to 33.59 fold. However, resistance declined significantly when the Thia-SEL strain reared for the next five generations without exposure to thiamethoxam. Compared to the laboratory susceptible reference strain (Lab-susceptible), the Thia-SEL strain showed cross-resistance to imidacloprid. Synergism tests revealed that *S*,*S*,*S*-tributylphosphorotrithioate (DEF) and piperonyl butoxide (PBO) produced synergism of thiamethoxam effects in the Thia-SEL strain (2.94 and 5.00 fold, respectively). In addition, biochemical analyses revealed that the activities of carboxylesterase (CarE) and mixed function oxidase (MFO) in the Thia-SEL strain were significantly higher than the Lab-susceptible strain. It seems that metabolic detoxification by CarE and MFO was a major mechanism for thiamethoxam resistance in the Thia-SEL strain of house flies. The results could be helpful in the future to develop an improved control strategy against house flies.

## Introduction

Sanitation and hygiene are the basic measures in the management of house flies, *Musca domestica* L., in and around livestock facilities. These measures could be applied by managing or eliminating the animal feces which play a significant role in the breeding of house flies. The subsequent measure is the use of insecticides, which is rather a difficult approach in part because house flies have the ability to develop resistance to insecticides used for their control [1, 2]. However, in contrast to insecticides which are applied as residual sprays for premises treatment, the chances of resistance development are low to those applied as baits [3].

Neonicotinoids baits are currently the fastest-growing class of insecticides and are replacing conventional insecticide baits used in the management of house flies [4]. Insecticides in the neonicotinoid class (e.g., imidacloprid, thiamethoxam) are synthetic derivatives of nicotine, an alkaloid compound present in the leaves of many plants. These insecticides exert their toxic effects via interactions with nicotinic acetylcholine receptors of the insect nervous system and are thus used extensively in the management of insect pests of agricultural and public health importance [5]. Thiamethoxam is the first commercial insecticide from the subclass thianicotinyl of neonicotinoids, which is usually used as a seed, foliar and soil treatment in agricultural operations, and as a bait against house flies [4].

Insecticide resistance is a worldwide problem which hinders the success of insect pest control programs. Few reports on thiamethoxam resistance and cross-resistance to other neonicotinoids have been reported in *Bemisia tabaci* (Gennadius) [6], *Frankliniella occidentalis* (Pergande) [7], *M*. *domestica* [4] and *Leptinotarsa decemlineata* (Say) [8]. According to reports, enhanced activities of cytochrome P-450 and carboxylesterase enzymes have been found to be associated with thiamethoxam resistance in some insect pests [7, 9]. Although, thiamethoxam, in Pakistan, has not yet been included in the management plans for house flies at livestock facilities, it is being extensively used on different crops for the management of different insect pests [10]. A recent report from Pakistan [11] revealed that most of the regional dairy producers practiced crop farming along with the animal farming around their dairy facilities. It was further reported that the use of insecticides from various classes including thiamethoxam on different crops around the dairies. Resultantly, there could be chances of house flies exposure to thiamethoxam during foliar applications.

Therefore, it is valuable to assess the risk of resistance development, resistance mechanism, and develop a proactive resistance management plan before time. The aim of the present study was to survey house flies resistance to thiamethoxam from different localities in the Punjab province, Pakistan. The study was further aimed to characterize thiamethoxam resistance risk in house flies by resistance selection pressure in the laboratory, cross-resistance to other insecticides and biochemical mechanisms. The results would be helpful in the future to develop a proactive and an improved control strategy against house flies.

## Materials and Methods

### Ethics statement

No specific permit was required to collect house fly samples from the dairy farms as these were privately owned and collection was made merely by speaking with the private owners. Since, the house fly is not an endangered species; no permission was required from any concerned authority in Punjab, Pakistan.

### Insects

Adult male and female house flies were collected (≈250 per site) from the dairies located in eight localities of Punjab, Pakistan: Sialkot (32.4972° N, 74.5361° E), Lahore (31.5497° N, 74.3436° E), Toba Tek Singh (30.9667° N, 72.4833° E), Multan (30.1978° N, 71.4697° E), Jhang (30.5833° N, 71.6500° E), DG Khan (30.0500° N, 70.6333° E), Bahawalpur (29.3956° N, 71.6836° E) and Rahim Yar Khan (28.4200° N, 70.3000° E). Each of the selected dairy farms was at least 50 km apart, and used different insecticides from all the classes like organochlorine, organophosphate, carbamate, pyrethroid and new chemicals for the management of different dairy pests [1], but none of the dairies used thiamethoxam. However, the selected dairy farms were surrounded by different crops like cotton, rice, maize, sugarcane, wheat, vegetables or fodders where thiamethoxam had been used for the management of different insect pests. A laboratory susceptible (Lab-susceptible) reference strain [1] was collected from an area of very low chemical use and maintained in the laboratory for more than 50 generations without any chemical exposure. The collected flies were reared in the laboratory as described previously [1] and maintained at 25±2°C, 65±5% RH and 12 L: 12 D period.

### Chemicals

Technical grade insecticides used in bioassays were: imidacloprid (95.3% AI), spinosad (90% AI) and thiamethoxam (97.7% AI). Two enzyme inhibitors *S*,*S*,*S*-tributylphosphorotrithioate (DEF; Sigma Ltd, UK), an esterase specific inhibitor, and piperonyl butoxide (PBO; Sigma Ltd, UK), an inhibitor of cytochrome P450 monooxygenases and of esterases [7] were also used to assess the biochemical mechanism.

### Bioassays

The insecticides were serially diluted in technical grade acetone. A no choice feeding bioassay method was used to assess the toxicities of different insecticides as described previously [1], with some modifications:

Briefly, twenty 3–5-day-old female flies (Lab-susceptible strain or F1 of field strains) were introduced into plastic containers (250 ml) and provided water on a cotton wick (2 cm length), and three 3.0 g sugar cubes impregnated with a serial dilution (0.5 mL) of thiamethoxam or imidacloprid or spinosad or a solvent-only control. The solvent in sugar cubes was allowed to evaporate for 1 h before the introduction of flies into the containers [12]. Five to eight concentrations (causing >0% and <100% mortalities) were prepared as serial dilutions for each insecticide and each concentration was replicated three times (60 flies for each concentration). The experimental design was a completely randomized design and to ensure true replications of each insecticide tested, a fresh series of serial dilutions was prepared from new stock solution each time (i.e. 3 stock solutions for three replicates) [13]. Bioassays were conducted at 25 ± 2°C, 60 ± 5% RH and 12:12 (L/D) photoperiod. Final mortality was assessed at 72 h of post-exposure to insecticides and all ataxic flies were assumed dead.

For synergism bioassays, 3–5-day-old female house flies of the Lab-susceptible and Thia-SEL strains were exposed topically to the enzyme inhibitors DEF and PBO at sublethal dose of 10 μg per fly, 1 h before the insecticide treatment [14, 15]. After an hour, the treated flies were bioassayed with different concentrations of thiamethoxam as stated above.

### Selection experiment

The field collected strain from Lahore, showing the lowest level of resistance to thiamethoxam, was re-selected in the laboratory with thiamethoxam (97.7%) as described previously [15, 16] with some modifications:

Briefly, the no-choice feeding method was used for selection and bioassays as described above. Before starting the selection experiment, a preliminary evaluation of the field collected flies was made to have an idea about the lethal concentration (LC) values for the desired selection process (i.e. 70% mortality). These evaluations were made before each subsequent selection so as to maintain 70% mortality selection pressure. For the selection experiment, unmated house flies (n = 2000–2500), less than a day old, were exposed to thiamethoxam via sugar cubes (as stated above) for 5 consecutive generations (G1–G5). Mortality data were recorded after 72 h of the exposure and the survivors were used as the parents of the next generation.

To assess stability of thiamethoxam resistance, the thiamethoxam selected (Thia-SEL) strain was then reared for the next 5 generations (G6–G10) without thiamethoxam exposure. This strain was assayed again with thiamethoxam at G11 to determine LC_50_ value, after which the rate of decrease or increase was calculated [17] as follows:

$$Rate\text{ of change in resistance (DR)}=[log(finalLC_{50})-log(initialLC_{50})/n]$$

Where, *n* is the number of generations reared without thiamethoxam exposure.

### Enzyme analyses

Analyses were done by using 3-5-day old female house flies. Individual female house flies were separated and homogenized in 1 ml of sodium phosphate buffer (0.1M; pH 7.8) at 4°C. The homogenates were centrifuged at 10,000×g at 4°C for 10 min, and large fragments were then removed [18]. The supernatant was used immediately for assaying the activities of carboxylesterase (CarE), mixed function oxidase (MFO), and glutathione *S*-transferase (GST) by using the methodologies of Gao et al. [7] for CarE and MFO, Yang et al. [19] for the determination of GST activity, and Bradford [20] for measuring total proteins.

### Statistical Analyses

All the mortality data were analyzed by Probit analysis [21] using the software SPSS (version 10.0) to determine the median lethal concentrations (LC_50_) and their 95% confidence intervals (CIs). LC_50_ values of the respective bioassays were considered significantly different on the basis of non-overlapping of 95% CIs [22]. Statistical comparison of enzyme activities in the Thia-SEL and Lab-susceptible strains were assessed using student t-test at a P value threshold of 0.05.

## Results

### Toxicity of thiamethoxam to different strains of house flies

The results of insecticidal bioassays revealed varying levels of resistance to thiamethoxam in different house fly strains when compared with the Lab-susceptible reference strain (Table 1). Of eight field strains of house flies tested against thiamethoxam, the strain from RY Khan showed the highest level of resistance (20.13 fold) followed by the Multan (18.65 fold), DG Khan (16.88 fold), Bahawalpur (16.08 fold), Sialkot (13.50 fold) and Jhang (15.26 fold) strains. However, the toxicity of thiamethoxam in the above field strains was statistically at par based on overlapping of 95% CIs. Whereas, the strain from Lahore showed the lowest level of resistance (7.66 fold) followed by the Toba Tek Singh strain (8.68 fold), both of the strains were statistically at par (overlapping 95% CIs) but significantly different from rest of the field strains (non-overlapping 95% CIs; Table 1).

**Table 1:** Toxicity of thiamethoxam to the laboratory susceptible and field strains of house flies.

Strain	n*	LC_50_ (95% CI)(μg/ml)	Fit of probit line				RR**
			Slope (SE)	χ^2^	df	*p*	
Lab-susceptible	420	2.49 (2.12–2.93)	2.33 (0.20)	2.80	4	0.59	
Sialkot	420	33.61 (23.17–59.56)	1.31 (0.18)	2.02	4	0.73	13.50
Lahore	420	19.08 (14.05–29.07)	1.26 (0.16)	1.92	4	0.75	7.66
Toba Tek Singh	480	21.62 (16.94–28.85)	1.37 (0.14)	2.56	5	0.76	8.68
Multan	480	46.45 (35.38–64.87)	1.23 (0.13)	3.73	5	0.58	18.65
Jhang	480	37.99 (29.52–51.05)	1.27 (0.13)	3.54	5	0.61	15.26
DG Khan	480	42.03 (31.99–58.44)	1.19 (0.12)	4.65	5	0.46	16.88
Bahawalpur	540	40.03 (31.08–53.88)	1.28 (0.11)	5.46	6	0.48	16.08
RY Khan	480	50.12 (39.74–65.04)	1.38 (0.13)	6.13	5	0.29	20.13

*number of flies used in bioassays

**resistance ratio

### Selection experiment

Selection of the field strain with thiamethoxam in the laboratory for five consecutive generations (G1–G5) revealed that the selection had a marked effect on the development of resistance (Table 2). The bioassays at G6 revealed that the LC_50_ values increased from 19.08 to 83.64 μg/ml, along with the RR values from 7.66 to 33.59 fold when compared with the lab-susceptible strain. The Thia-SEL strain was reared from G6–G10 without exposure to thiamethoxam and bioassayed again at G11 which revealed that the resistance to thiamethoxam was unstable (RR dropped from 33.59 fold to 21.85 fold) in the selected strain (Table 2).

**Table 2:** Selection history of the field strain of house flies with thiamethoxam, and subsequent toxicity after four generations without thiamethoxam exposure.

Strain	n*	LC_50_ (95% CI)(μg/ml)	Fit of probit line				RR**
			Slope (SE)	χ^2^	df	*P*	
G1 (Field)	420	19.08 (14.05–29.07)	1.26 (0.16)	1.92	4	0.75	7.66
G2	420	27.49 (21.84–36.35)	1.57 (0.17)	2.59	4	0.62	11.04
G3	420	37.60 (29.42–51.68)	1.61 (0.19)	4.08	4	0.39	15.10
G4	420	50.04 (33.31–87.56)	1.70 (0.18)	7.69	4	0.10	20.10
G5	420	76.58 (51.58–85.06)	1.70 (0.19)	4.65	4	0.32	30.76
G6 (Thia-SEL)	420	83.64 (76.46–108.93)	1.47 (0.16)	6.30	4	0.17	33.59
G 11	420	54.41 (43.82–69.71)	1.56 (0.17)	4.65	4	0.32	21.85

*number of flies used in bioassays

**resistance ratio

### Cross-resistance to imidacloprid and spinosad

The field strain was bioassayed, before the selection experiment at G1, against imidacloprid and spinosad with the resulting LC_50_ values 73.91 and 4.63 μg/ml, respectively (Table 3). The field strain was again bioassayed against both of the insecticides after the selection experiment with thiamethoxam at G6 which revealed significant cross-resistance to imidacloprid (LC_50_ increased 2.20 fold; non-overlapping 95% CIs). However, no significant increase in the LC_50_ value was observed for spinosad at G6 when compared with the value at G1 (overlapping 95% CIs).

**Table 3:** Cross-resistance analyses of the field collected strain of house flies to imidacloprid and spinosad after selection with thiamethoxam.

Strain	Insecticide	*n*	LC_50_ (95% CI)(μg/ml)	Fit of probit line				CR*
				Slope (SE)	χ^2^	df	*p*	
Field (G1)	Imidacloprid	420	73.91 (56.82–104.48)	1.48 (0.17)	0.52	4	0.97	
Thia-SEL (G6)	Imidacloprid	420	165.60 (135.12–207.89)	1.72 (0.17)	3.84	4	0.42	2.20
Field (G1)	Spinosad	480	4.63 (3.79–5.76)	1.71 (0.14)	3.56	5	0.61	
Thia-SEL (G6)	Spinosad	420	5.52 (3.75–5.79)	1.64 (0.16)	0.43	4	0.98	1.19

*cross-resistance ratio

### Synergism test and metabolic enzyme activities

The synergistic effects of DEF and PBO with thiamethoxam against the Lab-susceptible and Thia-SEL strains are shown in the table 4. A significant synergism was observed for both of the enzyme inhibitors (SR = 2.94 fold for DEF and 5.00 fold for PBO) in the Thia-SEL strain, but there was a negligible effect in the Lab-susceptible strain. The LC_50_ values of the field strain in comparison to the Lab-susceptible strain in the presence of DEF and PBO were higher by 14 fold and 8 fold, respectively, suggesting incomplete reversion of resistance to thiamethoxam. The biochemical analyses revealed that the CarE and MFO activities in the Thia-SEL strain were significantly higher than that in the Lab-susceptible strain (p<0.05; Table 5).

**Table 4:** Synergistic effect of enzyme inhibitors on the toxicity of thiamethoxam against house flies strains.

Strain	Compound	*n*	LC_50_ (95% CI)(μg/ml)	Fit of probit line				SR*
				Slope (SE)	χ^2^	df	*p*	
Lab-susceptible	Thiamethoxam	420	2.49 (2.12–2.93)	2.33 (0.20)	2.80	4	0.59	
	+DEF	420	2.38 (1.99–2.81)	2.12 (0.19)	2.88	4	0.57	1.04
	+PBO	420	2.04 (1.71–2.43)	2.08 (0.18)	2.21	4	0.69	1.22
Thia-SEL (G6)	Thiamethoxam	420	83.64 (76.46–108.93)	1.47 (0.16)	6.30	4	0.17	
	+DEF	420	28.49 (24.16–33.40)	2.41 (0.21)	5.20	4	0.26	2.94
	+PBO	420	16.73 (11.43–23.77)	2.09 (0.18)	7.77	4	0.10	5.00

*SR, synergism ratio calculated as the toxicity of thiamethoxam alone divided by the toxicity of thiamethoxam with DEF or PBO

**Table 5:** Metabolic enzyme activities in the Lab-susceptible and Thia-SEL strains of house flies.

Strain	CarE		MFO	
	Activity ± SE nmol min^-1^ mg^-1^	Ratio*	Activity ± SE pmol min^-1^ mg^-1^	Ratio*
Lab-susceptible	156.53 ± 3.37	1.00	38.10 ± 2.58	1.00
Thia-SEL	235.40 ± 3.61**	1.50	73.53 ± 2.45**	1.93

*enzyme activity in the Thia-SEL/ enzyme activity in the Lab-susceptible strains

**significantly different by applying student t-test (p<0.05)

## Discussion

New insecticides with the novel mode of action are increasingly difficult and costly to develop; therefore, it is essential to retain the efficacy of pre-existing insecticides through the development of resistance management strategies. The current status of insecticide resistance in field strains and underlying mechanisms of resistance, if known, could help to design effective resistance management strategies. The present study fulfills a part of this need by describing the level of resistance to thiamethoxam in different field strains of house flies in Punjab, Pakistan. The study was further aimed to study the potential of resistance development, cross-resistance to other insecticides and possible biochemical mechanisms of resistance to thiamethoxam in house flies. The results revealed that house flies from different areas showed varying levels of resistance to thiamethoxam. Although, none of the dairy farms used thiamethoxam for the control of house flies, neonicotinoids were being extensively used around the dairies for the management of different insect pests like aphids, jassids and whiteflies (personal communications). Therefore, it could be probable that house flies were indirectly exposed to residues from around the dairies and/or their past exposure to insecticides applied in dairy farms resulted in resistance against thiamethoxam and cross resistance to other insecticides. Previously thiamethoxam resistance pests have been reported in house flies from Danish livestock farms [8], but this is the first case from Pakistan.

After continuous selection in the laboratory for five consecutive generations, resistance to thiamethoxam increased from 7.66–33.59 folds, which revealed that the selection had a marked effect on resistance development in the selected strain. However, the potential to develop resistance to thiamethoxam could be associated with the insect species in question, genetic background, selection intensity and application history. For example, *F*. *occidentalis* from China [7] could develop only 15.1 fold resistance to thiamethoxam after 55 generations of selection, whereas *B*.*tabaci* from Israel [6] showed 100 fold resistance after 20 generations of selection with thiamethoxam. In the present study, there was a significant decline in the RR values (RR declined from 33.59 to 21.85 fold) when the selected strain was reared for the next five generations (G6–G10) without exposure to insecticides. This decline in resistance suggests that the allele (s) responsible for resistance to thiamethoxam might be unstable or the fitness cost might be high for maintaining the resistant allele(s) [15, 23]. In addition, the fitness studies of the Thia-SEL strain are under investigations in our laboratory. Nevertheless, the results of the present study revealed that the field strain can develop a high level of resistance to thiamethoxam, and the judicious use of thiamethoxam will be needed to retain its efficacy for long.

In the present study, the field strain (Thia-SEL) after selection with thiamethoxam showed significant cross-resistance to imidacloprid when compared with the strain before selection at G1. It shows that their use as alternatives for each other should be omitted. Such type of cross-resistance between thiamethoxam and imidacloprid has already been reported in different insect pests like *L*. *decemlineata* [24], *B*. *tabaci* [9, 25, 26] and *M*. *domestica* [8]. No cross-resistance between thiamethoxam and spinosad was observed in the present study which might provide an opportunity to use these products as alternates. However, there might be some risks in using spinosad and thiamethoxam in rotation or alternates since both insecticides are nicotinic activators [8] which may eventually result in resistance problems in the future.

To study the mechanism of thiamethoxam resistance in the Thia-SEL strain, synergism tests were conducted using the enzyme inhibitors PBO and DEF. The results revealed a significant decline in the LC_50_ values with synergism ratios 5 and 2.94 fold with PBO and DEF, respectively, suggesting that metabolic detoxification was involved in the development of thiamethoxam resistance. In addition, biochemical analyses revealed that the activities of CarE and MFO in the Thia-SEL strain were significantly higher than the Lab-susceptible strain. The both in vivo synergism tests and in vitro metabolic enzyme activities suggest that the increased activities of CarE and cytochrome P-450 enzyme were responsible for thiamethoxam resistance in the selected strain of house flies. These results are in agreement with previous reports on the mechanism of thiamethoxam resistance in different insect pests. For example, in *F*. *occidentalis* [7] and *B*. *tabaci* [9] thiamethoxam resistance have been found due to increased activities of cytochrome P-450 and CarE. Moreover, incomplete reversion of resistance to thiamethoxam suggest that other mechanisms like modification of target sites may also involve along with metabolic detoxification in evolving resistance to thiamethoxam in the selected strain of house flies.

In conclusion, the study demonstrates the presence of varying level of resistance to thiamethoxam in different field strains of house flies. The laboratory selection showed the potential of house flies to develop a high level of thiamethoxam resistance under continuous selection pressure. The Thia-SEL strain also showed cross-resistance to imidacloprid, but no obvious cross-resistance to spinosad. In addition, enhanced activities of CarE and cytochrome P-450 enzymes are seem to be associated with thiamethoxam resistance. Instability of thiamethoxam resistance suggests that the toxicity of thiamethoxam to house flies could be restored by releasing selection pressure. This will ultimately delay the development of resistance, and result in enhanced life of the product. Although thiamethoxam has not yet been included in the management plans of house flies in Pakistan, the results of the present study could be helpful in the development of a proactive resistance management plan whenever thiamethoxam resistance crisis become severe in the future. Moreover, regular resistance monitoring and studies on inheritance analyses of thiamethoxam resistance will be needed in the future to develop an improved control strategy against house flies.
